# Differential Gene Expression Between Polymorphic Zooids of the Marine Bryozoan *Bugulina stolonifera*

**DOI:** 10.1534/g3.120.401348

**Published:** 2020-08-28

**Authors:** Kira A. Treibergs, Gonzalo Giribet

**Affiliations:** Museum of Comparative Zoology, Department of Organismic and Evolutionary Biology, Harvard University, Cambridge, MA 02138

**Keywords:** avicularia, RNA-sequencing, *de novo* transcriptome, heterozooids

## Abstract

Bryozoans are a diverse phylum of marine and freshwater colonial invertebrates containing approximately 6,300 described living species. Bryozoans grow by budding new physiologically connected colony members (zooids) from a founding individual that forms from a metamorphosed larva. In some species these zooids come in different shapes and sizes and are specialized to serve different tasks within the colony. A complex interaction of genotype, environment, and developmental pathway shapes zooid fate, however, the specific mechanisms underlying the establishment of this division of labor remain unknown. Here, the first characterization of differential gene expression between polymorphic zooids of a bryozoan colony is presented. The development of different zooid types of lab-cultured *Bugulina stolonifera* colonies including feeding autozooids, avicularia (derived non-feeding zooids that are homologous to feeding autozooids but shaped like a bird’s beak), and rhizoids (a branching network of non-feeding anchoring zooids) was explored using RNA sequencing, *de novo* transcriptome assembly, and differential gene expression analyses. High throughput sequencing of cDNA libraries yielded an average of 14.9 ± 1.3 (SE) million high-quality paired-end reads per sample. Data for the first *de novo* transcriptome assemblies of *B. stolonifera* and the first characterization of genes involved in the formation and maintenance of zooid types within a bryozoan colony are presented. In a comparison between autozooid and avicularium tissues, 1,097 significant differentially expressed genes were uncovered. This work provides a much-needed foundation for understanding the mechanisms involved in the development of polymorphic zooids and the establishment of division of labor in bryozoans.

Division of labor as a means of increasing biological efficiency is a commonly observed theme in many of the major evolutionary transitions of life. This includes the transition from single celled to multicellular organisms, from asexual populations to sexual populations, and from solitary organismal associations to colonial organisms (Szathmáry and Smith 1995; Simpson 2012, Hiebert *et al.* 2020). Of all the metazoan phyla with colonial animals, only the Cnidaria, Bryozoa, and Chordata have representatives with division of labor among asexually budded and physiologically connected colony modules, or zooids. This phenomenon, known as zooid polymorphism, is characterized by colonies having discontinuous variation in the anatomy of zooids, associated with division of labor and resource sharing within colonies (Harvell 1994).

Bryozoans are a fascinating phylum of aquatic metazoans that form colonies with an exceptionally high degree of colony polymorphism (Waeschenbach *et al.* 2012). All bryozoan colonies are composed of autozooids, the basic feeding and reproductive zooid form, that constitutes a feeding unit (polypide) that captures food particles with a ring of ciliated tentacles (lophophore). The polyp is housed in a skeletal unit called zooecium (Figure 1). Many species of bryozoan have different types of asexually budded non-feeding zooids, known as polymorphic zooids or heterozooids, that have distinct morphology and structures from autozooids. In the order Cheilostomata, these polymorphic zooids are quite common, and range in form and function from defense, embryo brooding, locomotion, structural support, and colony attachment. All polymorphic zooids are incapable of feeding and instead, obtain nutrients from the funicular system, a tissue extension which interconnects all zooids via pores in zooecium walls in gymnolaemates, one of the three major bryozoan clades, which contains the order Cheilostomata (Mukai *et al.* 1997; Carter *et al.* 2010b; Schwaha *et al.* 2020). One of the most commonly observed polymorphic zooids found in cheilostome colonies is the avicularium, a derived module that is homologous to the feeding autozooid (Carter *et al.* 2011). Avicularia have often been presumed to be defensive, as avicularia of certain species have been observed trapping micro predators (Kaufmann 1968; Winston 1986, 1991), however empirical evidence supporting this hypothesis in most bryozoan species is largely lacking (Winston 1984; Carter *et al.* 2010a).

**Figure 1:**
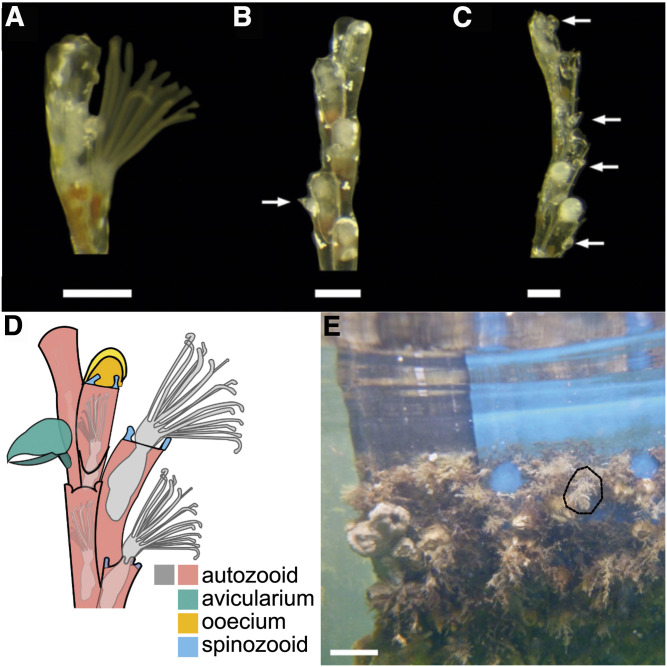
*Bugulina stolonifera* colony details including branch tips (A, B, C, D) and whole colonies in the field (E). A) An autozooid with the lophophore extended, B) four and a half autozooids and one avicularium (indicated by arrow) on the fourth most distal zooid, C) a colony branch tip with five autozooids bearing two round embryo-brooding chambers with opaque developing larvae (ovicells) and several avicularia (indicated by arrows), and D) colony branch tip diagram depicting common zooid types. E) Representative fouling community found in Eel Pond, Falmouth, MA, with one colony of *B. stolonifera* circled (dashed line). Colony branches (A, B, C, D) are oriented such that the youngest zooids (branch tips) are at the top of the figure. Colonies (A, B, C) were cultured from larvae in the laboratory. Scale bar for A, B, and C is 500 *μ*M, and scale bar for D is 5 cm.

Although they possess vestiges of a polypide, avicularia are incapable of feeding (Hyman 1959; Silén 1977; Carter *et al.* 2010b 2011). Instead, they must obtain nutrients from neighboring autozooids via the funicular system (Mukai *et al.* 1997). Although morphologically diverse, most avicularia are characterized by having an enlarged, hinged operculum-derived “mandible” that can be opened and shut with pairs of hypertrophied muscles (Winston 1984; Carter *et al.* 2011; Schack *et al.* 2019).

During colony development, avicularia and all other zooid types form at particular budding sites in the epithelial layer of the body wall of the parent zooid. These budding sites consist of condensations of cells that eventually will differentiate into daughter zooids (Mukai *et al.* 1997; Lidgard *et al.* 2011). Newly budded daughter zooids are connected to their parent zooids by a branching series of cords (the funiculus) and a nerve network, resulting in a colony that is highly functionally integrated (Mukai *et al.* 1997; Carle and Ruppert 1983; Lidgard *et al.* 2011; Schack *et al.* 2019). How the zooidal fate of the newly budded daughter zooid is determined, however, is poorly understood. Evidence suggests that a complex interaction of genotype, environment, and developmental pathways shapes anatomy at both the zooidal and colonial level (Harvell 1994), however the developmental, genetic, and epigenetic basis of this variation remains largely unknown (Lidgard *et al.* 2011; Schack *et al.* 2019). In this study, these questions are explored using the autozooids and two heterozooids (the bird’s beak-shaped avicularia and root-like substrate attachment zooids called rhizoids) of the arborescent bryozoan *Bugulina stolonifera* (Ryland 1960) (Figure 1).

Cheilostomes such as *B. stolonifera* have an extensive and relatively well-preserved fossil record and about 4,800 described extant species that inhabit almost every benthic marine habitat (Brood 1998; Winston 2010; Waeschenbach *et al.* 2012; Bock and Gordon 2013; Zhang 2013; WoRMS Editorial Board 2019). In addition, cheilostomes make excellent laboratory study organisms as they have relatively short generation times, rapid growth rates, clearly discernible boundaries of colony modules, high levels of colony integration, regenerative capabilities, and amenability to laboratory culture (Hughes 2005; Winston 2010; Sköld and Obst 2011). Bryozoans in the genera *Bugula* and *Bugulina* have lecithotrophic coronate larvae that are easily obtained from field-collected colonies (Dahms *et al.* 2007; Temkin 2014) and can be cultured to adulthood for multiple generations (Johnson 2010).

By controlling the source of food and limiting the growth of colony epibionts in laboratory culture, researchers can limit possible environmental sources of contamination in samples cultured for future molecular analysis. Contamination by organisms closely associated with field-collected bryozoan colonies is a well-known source of sequencing error, and has been one of the main challenges in the reconstruction phylogenies involving bryozoans (Waeschenbach *et al.* 2012; Nesnidal *et al.* 2013). In this study, we investigated the genetic basis for the establishment of division of labor among zooids in a bryozoan colony, by comparing differential gene expression between three different zooid types in *B. stolonifera* across two stages of colony development (from bud to mature form).

## Materials and Methods

A novel technique to pool zooids dissected from lab-cultured bryozoan colonies was used, in order to get enough tissue for high-quality RNA sequencing. The issue of sequence contamination was addressed in two important ways, first, by culturing bryozoan colonies in the laboratory from a larval stage, thus minimizing the growth of fouling epibionts, and second, by generating a transcriptome of the bryozoan’s food source and filtering out any food contaminant sequencing reads from all bryozoan samples prior to transcriptome assembly.

### Bryozoan collection and culturing

Approximately 150 colonies of the arborescent bryozoan *Bugulina stolonifera* were collected off the sides of docks in Eel Pond, in Falmouth, Massachusetts on the mornings of July 1, 2015 and June 17, 2016 (Fig. S1). Specimens were transported in seawater on ice to the laboratory in Cambridge, MA, spending no more than three hours in transit. Colonies were maintained in Eel Pond seawater (EPSW) with aeration, in a 22° growth chamber in complete darkness. Unfiltered EPSW was used because bryozoan survival rates were higher, compared to filtered EPSW (personal observation). To decrease the likelihood of contamination from organisms in unfiltered water, EPSW was aged in the dark for 1 week (Johnson 2010).

Approximately 20 hr later, colonies were exposed to bright light (from a fiber optic microscope lamp) in order to trigger the release of larvae, which are positively phototactic. Larvae were concentrated using spot illumination and transferred into 1 mL drops of water on 45 mm diameter acetate discs (cut from transparent film, VWO100C-BE) with five larvae per water droplet, where they were covered and left in darkness. After 24 hr, the discs were monitored for permanent attachment of early metamorphs and any remaining unattached larvae were removed. In conditions where multiple larvae attached to the same acetate disc, metamorphs were removed such that only one remained per disc. Discs were then placed with metamorphs into 45 mm petri dishes, and vertically suspended (to prevent the accumulation of food or waste on the growing colonies) in 150 mL beakers containing 125 mL of EPSW (Fig. S2) and placed on a gently rocking orbital shaker in a 22° Culture chamber, with a 12 h light / 12 h dark cycle (Johnson 2010).

Bryozoan colonies were fed the cryptophyte *Rhodomonas salina* (Wislouch) Hill & Wetherbee, 1989 (Bigelow Labs, Strain CCMP1319) daily, at a concentration of 10,000 cells / mL. The *R. salina* cultures were maintained in f/2 -Si media by inoculating fresh culture media with cultured cells in exponential growth phase approximately every four days. Every other day, prior to feeding, colonies were cleaned, monitored for health, and water was replaced. Cleaning involved brushing accumulated waste from the colony and acetate disc with a soft paintbrush and replacing soiled petri dishes and beakers for clean substitutes, containing fresh EPSW. Health monitoring involved observing each colony closely under a dissecting microscope, looking for evidence of active feeding (lophophores everted in feeding posture), evidence of past feeding (zooids with red-pigmented guts, evidence of *R. salina*) and continued evidence of colony growth (new zooid growth zones and bifurcations at branch tips and rhizoid extension, bifurcation and autozooid formation).

A total of 23 colonies were cultured from larvae that metamorphosed on July 2^nd^, 2015. Each grew to over 60 bifurcations within one month, and polymorphic zooids started appearing as soon as five days post-metamorphosis (Fig. S3). Thirty colonies were cultured in 2016 that grew to over 60 bifurcations within one month with avicularia appearing in some colonies as early as four days post-metamorphosis. In the spring of 2016, 14 samples were prepared for RNA-sequencing, and in the spring of 2017, five additional samples were prepared (Table S1).

### Sample preparation and mRNA Extraction

**Figure 2:**
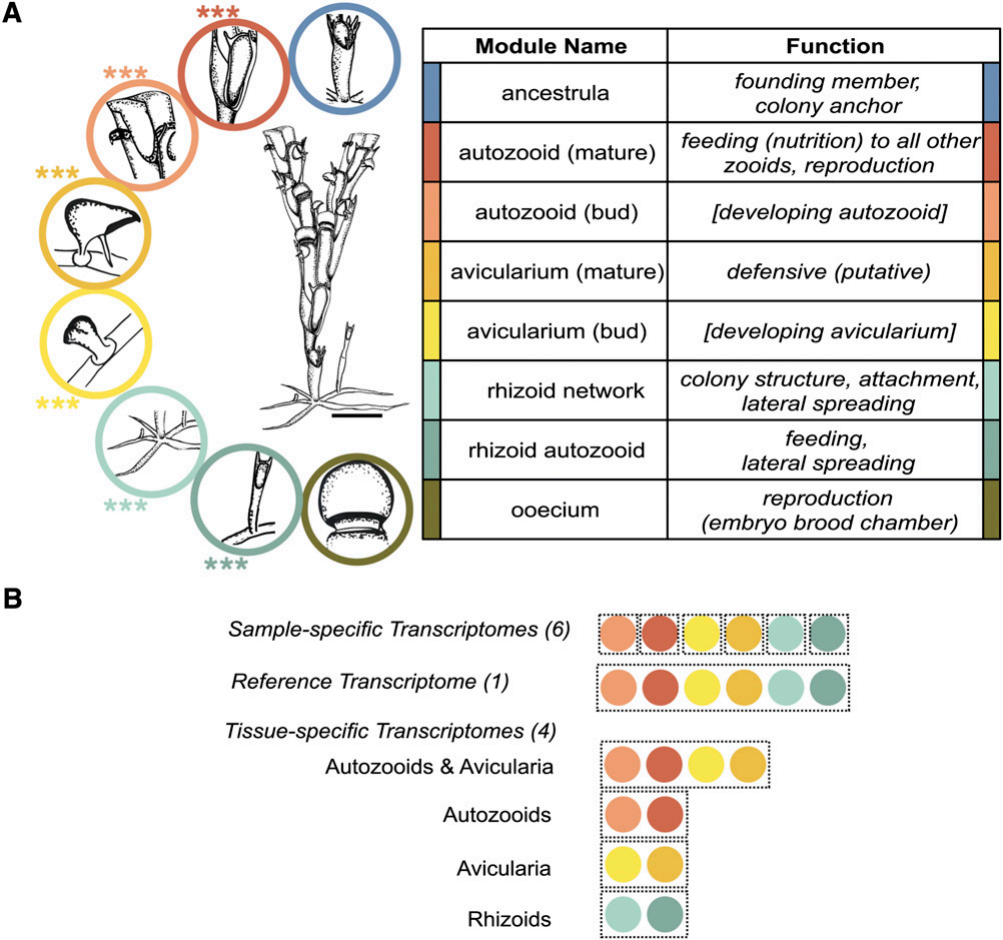
A) *Bugulina stolonifera* colony and its polymorphs. Adapted from Ryland, 1960 (Fig. 6, pg. 9). Scale bar is 500 µm. Asterisks indicate zooids that were dissected for RNA extraction (three biological replicates each), for a total of 18 samples. B) Eleven *B. stolonifera* transcriptomes were assembled using the software, Trinity (Haas *et al.*, 2013) resulting in six sample-specific, one reference, and four tissue-specific assemblies. Tissue-specific assemblies include: autozooids (mature autozooids and autozooid buds), avicularia (mature avicularia and avicularium buds), and rhizoids (rhizoid stolons and rhizoid autozooids). Each sample comprised of three biological replicates.

Three zooid types (autozooids, avicularia and rhizoids) were dissected at two different developmental stages (autozooid bud and mature autozooid, avicularium bud and mature avicularium, and rhizoid network and rhizoid autozooid) from lab-cultured *B. stolonifera* colonies (Figure 2A), in preparation for mRNA extraction, sequencing, and downstream differential gene expression analysis.

In order to harvest sufficient tissue to extract mRNA and construct cDNA libraries from bryozoan samples, multiple zooids from a single colony (assumed to be one genetic unit) were pooled together into one sample. Three biological replicates were collected for each tissue type and developmental stage, for a total of 18 samples (Figure 2). One sample of each set of three biological replicates came from the same genetic individual, and the remaining biological replicates each came from independent colonies. A sample of the cryptophyte *R. salina* was also prepared for RNA extraction and subsequent RNA sequencing, so that any gene expression signals from food source contamination could be filtered out from true signal of bryozoan gene expression.

RNA extraction and cDNA library building methods are modified from the protocols of Fernández *et al.* (2014). *Bugulina stolonifera* colonies intended for RNA-extraction were fixed in RNA*later* stabilization solution (Invitrogen, AM7021) and stored at -20° prior to dissection. Colonies were not fed within 24 hr of dissection. Sterilized micro-dissection forceps were used to dissect individual zooids from colonies submerged in filtered seawater over ice, a process that could take up to several hours per colony. Immediately after dissection, individual zooids were transferred to Eppendorf tubes with ice-cold 500 μL Trizol (Invitrogen, 15596026), and flash frozen in liquid nitrogen. Due to their small size and the significant time it took to dissect individual avicularia, these were dissected in batches of ten and then pooled together, to reduce the time that bryozoan tissue spent at temperatures above freezing and thus minimizing any potential degradation of mRNA.

Approximately 150 mature avicularia were dissected from each colony and combined to make up a single mature avicularium sample. Due the limited number of avicularia buds present in a colony, every avicularium bud in the entire colony (approximately 30 avicularium buds per colony) was dissected and pooled into an avicularium bud sample. Mature autozooid samples included ten autozooids that were dissected from the region between the fifth and tenth most distal autozooids, characterized by having fully developed skeletal features and polypides. Autozooid bud samples included ten of the most distal two autozooids from colony branch tips, characterized by having rounded undifferentiated features lacking the visible polypide structure or the skeletal features of the fully-developed autozooids. Rhizoid network samples included the complete branching network of undifferentiated rhizoid stolons, which were removed from the base of the colony, and rhizoid autozooid samples included approximately five autozooids that originated directly from rhizoid stolons. No avicularia or ooecia were included in either developmental stage of the autozooid or rhizoid samples (Figure 2).

In order to prepare a sample of *R. salina* for RNA extraction, we centrifuged 5 mL of cryptophyte culture in logarithmic growth phase (approximately 2,500,000 cells in total) was centrifuged, and the supernatant removed. Cells were stored in 500 µL of Trizol at -80° until the time of extraction.

Immediately prior to RNA extraction, 10 µL of glycogen (Thermo Scientific, R0551) were added to each sample and mechanically homogenized tissue with a power drill fitted with a polypropelyne pestle (Sigma Aldrich, Z359947). RNA was extracted with 100 µL of 1-Bromo-3-chloropropane (BCP, Sigma Aldrich B9673), and the samples were centrifuged at 16,000 rcf for 15 min at 6° to isolate RNA in the aqueous layer. RNA was precipitated out of solution using 500 µL of isopropanol (Sigma Aldrich, I9516) and centrifuged at 16,000 rcf for 15 min at 4° into a pellet that was washed twice in 1 mL of 75% ethanol, centrifuged for 7,500 rcf for 5 min at 4°, and then re-suspended in 50 µL RNA storage solution (Invitrogen, AM7000) and 1 µL of RNAse inhibitor (Applied Biosystems, N8080119). Messenger RNA was isolated from total RNA via poly-A selection using the Dynabeads mRNA Purification Kit (Invitrogen, 61006) and stored the mRNA at -80°. As a quality control measure to ensure sufficient mRNA yield and quality for downstream analysis, mRNA concentration and quality of each sample were assessed via electophoresis using the Agilent 2100 Bioanalyzer System with an RNA 6000 Pico chip (5067-1513).

### Building and sequencing cDNA libraries

Individual cDNA libraries were constructed from mRNA samples using the integenX Apollo 324 System following the PrepX mRNA8 protocol. Using reagents and protocols from the Kapa Library Amplification kit (Kapa Biosystems, KR0408-v7.17), libraries were amplified and sample-specific adapters (PrepX RNA-Seq Index Primers) were added onto cDNA fragments for future multiplexing. Twelve cycles of PCR were completed for autozooid and rhizoid samples, which had a large amount of starting tissue, and 18 cycles of PCR were completed for avicularia samples, which had a smaller amount of starting tissue and required additional PCR cycles in order to yield sufficient quantity of cDNA for sequencing. The AMPure XP beads were used in the Apollo 324 system to clean the PCR products via the PrepX mRNA8 PCR Cleanup protocol. cDNA sample quality (library size distribution and concentration) was assessed via electrophoresis using the Agilent 2100 Bioanalyzer System with a High Sensitivity DNA chip (5067-4626). cDNA library concentration was quantified via qPCR using the Kapa Library Quantification Kit (Kapa Biosystems, KK2611), and the libraries were then pooled in equimolar concentration for multiplexed sequencing. Samples were sequenced as paired-end, 150 bp reads in a rapid run flowcell on an Illumina HiSeq 2500 at the Bauer Core Facility at Harvard University.

### Data sanitization

Prior to *de novo* transcriptome assembly, questionable sequencing reads (due to low quality, small size, and potential contamination) were removed from each bryozoan sample. First, the software Trimgalore (Martin 2011) was used to remove low-quality reads from the dataset (with a Phred score cutoff of 20, a minimum read length threshold of 36 bp, a stringency parameter of 1 for adapter sequence overlap, and a maximum allowed error rate of 0.1) and to trim away Illumina adapters sequences (used for multiplexing) from raw sequencing reads. Rcorrector (Song 2015) was then used to correct random sequencing errors.

FastQC was used to identify over-represented sequences, which were confirmed via GenBank (Clark *et al.* 2016) to be largely ribosomal RNA, and removed from the dataset. Further read contamination was removed using Bowtie2 (Langmead and Salzberg 2012) by mapping *B. stolonifera* reads to a database of bacterial, archaean, and eukaryotic ribosomal RNAs (SILVA small subunit (SSU) and large subunit (LSU) “Parc” databases, July 7, 2017) (Quast *et al.* 2013), along with all GenBank bryozoan rRNA sequences and *R. salina* nucleotide sequences from (accessed July 7, 2017).

To remove any additional sequencing reads from the bryozoan samples due to contamination from the cryptophyte food-source, a *de novo* transcriptome of *R. salina* was assembled using Trinity (Grabherr *et al.* 2011) and mapped all *B. stolonifera* reads to the *R. salina* transcriptome assembly using Bowtie2. Any read pairs where one or both reads mapped successfully to the *R. salina* transcriptome from the *B. stolonifera* dataset were then removed. FastQC (http://www.bioinformatics.babraham.ac.uk/projects/fastqc) was used to assess sequencing quality for each pair of raw reads pre-sanitization and post-sanitization, to ensure that only high-quality reads remained, that there was no remaining read contamination from Illumina adapters or food source, and that when blasted to NCBI, any overrepresented sequences that remained were identified as bryozoan mRNA or ‘no sequence identity’.

### Bryozoan transcriptome assemblies

After sanitization, eleven *de novo* transcriptomes were assembled using the software Trinity (Grabherr *et al.* 2011) with read normalization (Figure 2B). Six sample-specific assemblies were constructed for each zooid type at two stages of development (avicularium bud, mature avicularium, autozooid bud, mature autozooid, rhizoid stolon, and rhizoid autozooid), for future assessment of read coverage and assembly completeness by sample, as a quality control measure to ensure that each tissue sample contributed a complete transcriptome. Additionally, a reference transcriptome from all six sample types together was constructed, for a downstream comparison by genetic individual. Lastly, four additional zooid-specific assemblies were constructed to more clearly isolate a signal of tissue-specific expression in downstream differential gene expression analyses, focusing on four biologically relevant pairwise contrasts (Álvarez-Campos *et al.* 2019). The first contrast of focus is between autozooids and their derived homologs, and the remaining three contrasts between two developmental stages of our three zooids of interest, the avicularia (autozooid buds, mature autozooids, avicularium buds, and mature avicularia), autozooids (autozooids buds and mature autozooids), avicularia (avicularium buds and mature avicularia), and rhizoids (rhizoid stolons and rhizoid autozooids). Each sample contributing to a transcriptome assembly included three biological replicates.

In order to assess the extent to which the assembled transcripts represented the sequencing reads, the sanitized, forward and reverse reads were mapped separately back to each assembly using Bowtie2 and assessed the percent of proper *vs.* improper pairing (an improper pair is defined as when forward and reverse reads from the same read pair map to different contigs). The program BUSCO (v3) (Waterhouse *et al.* 2017) was also used to quantify assembly completeness by searching for a curated set of single copy orthologs present in all metazoans (*metazoa odb9* database, accessed March 15, 2019).

### Differential gene expression analysis

In order to prepare transcript count and normalized gene expression matrices for downstream differential expression analysis, RSEM (v 1.3.1) (Li *et al.* 2014) was used to quantify transcript abundance and align reads to the reference and tissue-specific assemblies. The Trinity downstream analysis program ‘PtR’ was used to compare biological replicates across samples, by constructing a correlation matrix for each sample in an assembly, and principal components plots, labeled by tissue and genetic individual. Next, the *R* Bioconductor package, *edgeR* (Robinson *et al.* 2010) was used to conduct pairwise comparisons of differential gene expression by sample type, for the reference assembly (comparing autozooid bud, mature autozooid, avicularium bud, mature avicularium, rhizoid network and rhizoid autozooid samples), autozooid assembly (comparing autozooid bud samples with mature autozooid samples), the avicularium assembly (comparing avicularium bud samples with mature avicularium samples), the rhizoid assembly (comparing rhizoid autozooid samples with rhizoid network examples), and the autozooid and avicularium assembly (comparing autozooid bud and mature autozooid samples with avicularium bud and avicularium samples). Genes were considered to be significantly differentially expressed (DE) if they were greater than or equal to a fourfold expression level with a false discovery rate (FDR) *p*-value < 0.001, a stringency cutoff that highlights transcripts with the greatest expression differences between zooids. Sample-specific expression for the reference assembly (comparing all six zooid types) was calculated from each pairwise comparison by averaging across replicates and summarizing the consistently up-regulated genes for each zooid type, using the Trinity script ‘pairwise_DE_summary_to_DE_classification.pl’ (Haas *et al.* 2013).

We then functionally annotated the lists of significantly differentially expressed genes (DEG) from each transcriptome using the software *Trinotate* (Bryant *et al.* 2017), by blasting predicted longest open reading frame peptides (predicted using *Transdecoder* [Haas *et al.* 2013]) and nucleotide transcripts from each assembly to the NCBI ‘NR’ database (with an E-value < 1e-5). To characterize the biological functions of the DE genes in each assembly, GO assignments were extracted from significantly DE transcripts and longest open reading frame peptides (by blasting to the SwissProt database with an E-value < 1e-5), conducted gene ontology enrichment analysis using the *R* Bioconductor package GoSeq (Young *et al.* 2010), and graphically summarized the GO terms with the program *Revigo* (Supek *et al.* 2011).

### Data availability

Raw sequence data have been accessioned to the NCBI SRA database under SRA project SRX7735396. A subset of field-collected and lab-cultured colonies from this project was fixed in ethanol and deposited in the Invertebrate Zoology collection of the Museum of Comparative Zoology and can be accessed on MCZbase (mczbase.mcz.harvard.edu) via accession numbers 2000356 and 2000886. Transcriptome assemblies and transcriptome annotations generated from this study can be publicly accessed on Harvard Dataverse (https://doi.org/10.7910/DVN/SDJZ4X). Supplemental material available at figshare: https://doi.org/10.25387/g3.12786395.

## Results

### Transcriptome assembly

**Figure 3:**
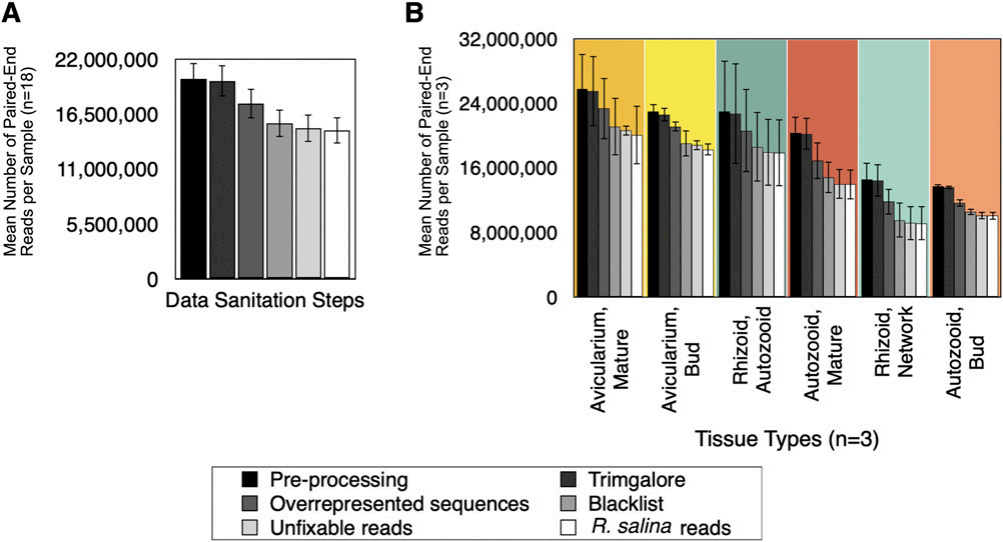
Sanitization of bryozoan sequences. Mean (± SE) number of paired-end reads is plotted for A) all samples pooled together (n = 18) and for B) individual samples (n = 3). Each sample went through four steps of filtering: removing adapter contamination and low-quality reads (Trimgalore), removing overrepresented sequences (as identified by FastQC), removing any reads that mapped to a bacterial, archaean, and eukaryotic ribosomal RNA databases (SILVA small subunit (SSU) and large subunit (LSU) “Parc” databases) along with all bryozoan rRNA and *R. salina* sequences from GenBank (accessed July 7, 2017), removing any reads with sequencing errors that were deemed ‘uncorrectable’ (Rcorrector), and removing any reads that mapped to the *R. salina* transcriptome.

High throughput sequencing of cDNA libraries yielded an average of 20.1 ± 6.2 (SE) million paired-end reads per sample, with 94% of bases having a quality score over 30 (Table 1). After filtering low quality reads from the assembly, removing reads with uncorrectable sequencing errors, removing possible ribosomal RNA contamination, and removing the 0.54–9.0% of those reads that mapped to the *Rhodomonas salina* transcriptome (Fig. S4, Table S2), an average of 14.9 ± 1.3 (SE) million paired-end reads remained (Figure 3). Eleven transcriptomes were assembled in total that had, on average, 158,258 ± 23,246 (SE) total ‘Trinity genes’ (in this case ‘Trinity gene’ is a category defined by the software Trinity as a cluster of similar transcripts), 249,576 ± 23,046 (SE) total transcripts, and 42.5% GC content (Table 2).

**Table 1:** Sequencing metrics for raw reads of *B. stolonifera* samples. Raw read counts by bryozoan tissue and replicate, as determined by the Illumina HiSeq 2500. Asterisks indicate the biological replicate that originated from the same genetic individual. ’PE Reads’ refers to paired end reads. A quality score of 30 represents *P* = 0.001

Sample Tissue	PE Reads	Bases with Quality > 30
	(#)	(%)
Autozooid, Bud-1	13,496,155	95.10
Autozooid, Bud-2	13,389,595	95.39
Autozooid, Bud-3*	14,172,054	93.67
Autozooid, Mature-1	22,899,047	95.08
Autozooid, Mature-2	21,689,242	95.59
Autozooid, Mature-3*	16,366,673	95.68
Avicularium, Bud-1	24,427.060	93.25
Avicularium, Bud-2	21,277,536	94.26
Avicularium, Bud-3*	23,257,584	92.68
Avicularium, Mature-1	20,466,757	93.03
Avicularium, Mature-2	34,397,850	93.85
Avicularium, Mature-3*	22,417,208	94.87
Rhizoid Network-1	10,976,828	94.10
Rhizoid Network-2	18,043,140	93.84
Rhizoid Network-3*	14,698,226	93.96
Rhizoid, Autozooid-1	34,129,055	97.10
Rhizoid, Autozooid-2	22,578,272	94.47
Rhizoid, Autozooid-3*	12,255,318	94.81
**Mean**	**20,052,089**	**94.49**
**SE**	**1,585,445**	**0.26**

**Table 2:** Trinity transcriptome assembly metrics for *B. stolonifera*. Six sample-specific assemblies were constructed including autozooid bud, mature autozooid, avicularium bud, mature avicularium, rhizoid network and rhizoid auto zooid (n = 3). Four tissue-specific assemblies were constructed including autozooids (autozooid buds and avicularia, n = 6), avicularia (avicularium buds and mature avicularia, n = 6), and rhizoids (rhizoid networks and rhizoid autozooids, n = 6) and autozooids & avicularia (autozooid buds, mature autozooids, avicularium buds, and mature avicularia, n = 12) and one reference assembly that included all samples (n = 18)

	*Stats based on all transcriptome contigs:*				*Stats based on longest isoform per gene:*			
Sample Tissue	Trinity Genes	Transcripts	GC	Contig N50	Contig N50	Median Contig	Average Contig	Total Bases
	(#)	(#)	(%)	(bp)	(bp)	(bp)	(bp)	(#)
Autozooid, Bud	57,794	160,847	41.65	1,138	1,061	360	649.94	37,562,715
Autozooid, Mature	174,411	218,229	41.50	779	499	293	462.37	80,502,834
Avicularium, Bud	103,528	144,512	43.36	841	530	274	454.97	47,102,299
Avicularium, Mature	148,534	246,836	43.07	1,232	564	291	472.64	70,203,415
Rhizoid Network	97,761	168,398	42.87	845	559	275	472.55	43,362,107
Rhizoid, Autozooid	95,153	209,128	42.43	1,192	854	284	536.28	51,028,728
Autozooids	157,357	325,134	41.43	1,096	617	617	495.53	77,974,357
Avicularia	191,557	297,736	43.42	1,106	478	478	445.44	85,327,712
Rhizoids	143,840	285,061	42.62	1,055	576	576	475.45	68,389,288
Autozooids & Avicularia	254,637	306,639	42.19	662	437	437	436.54	111,159,221
Reference	322,564	382,817	42.41	567	408	408	417.98	134,826,824
**Mean**	**158,258**	**249,576**	**42.5**	**955.7**	**598.5**	**286**	**483.6**	**73,403,591**
**SE**	12,246	23,046	0.2	68.2	58.3	2	19.1	8,957,786

**Figure 4:**
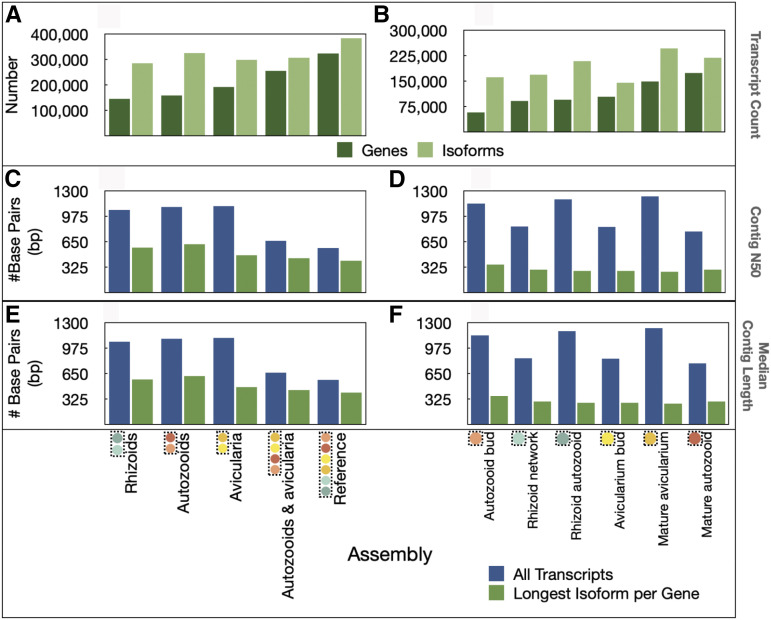
Transcriptome assembly metrics. Number of transcripts is plotted for each A) tissue-specific and B) sample-specific assembly. Contig N50 is plotted for each C) tissue specific and D) sample-specific assembly. Median contig length is plotted for each E) tissue-specific assembly and F) sample specific assembly.

For these transcriptomes, based on the longest isoform per gene, contig N50 values ranged from 408–1061 bp, median contig lengths ranged from 266–360 bp, and average contig lengths ranged from 418–650 bp. The reference transcriptome (containing three biological replicates of each sample) had the highest number of genes of all the assemblies, but had the smallest contig N50 value, median contig size, and average contig length (Figure 4).

**Figure 5:**
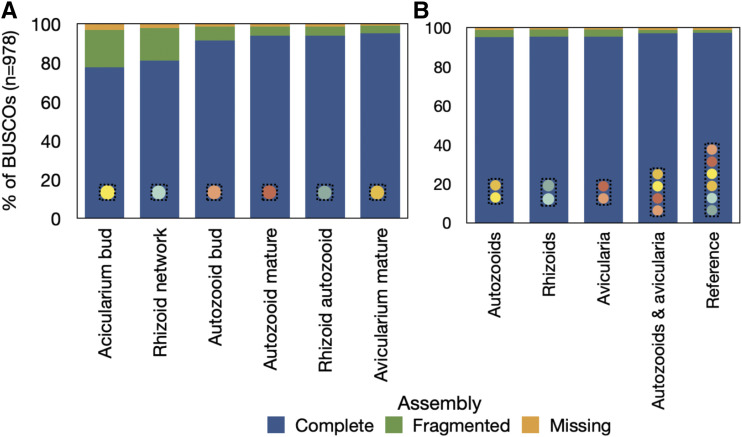
Assembly completeness as calculated for *B. stolonifera* A) sample-specific assemblies and B) tissue-specific assemblies, according to the BUSCO *metazoa odb9* database (accessed August 17, 2018) which contains a total of 978 curated single-copy gene orthologs.

**Figure 6:**
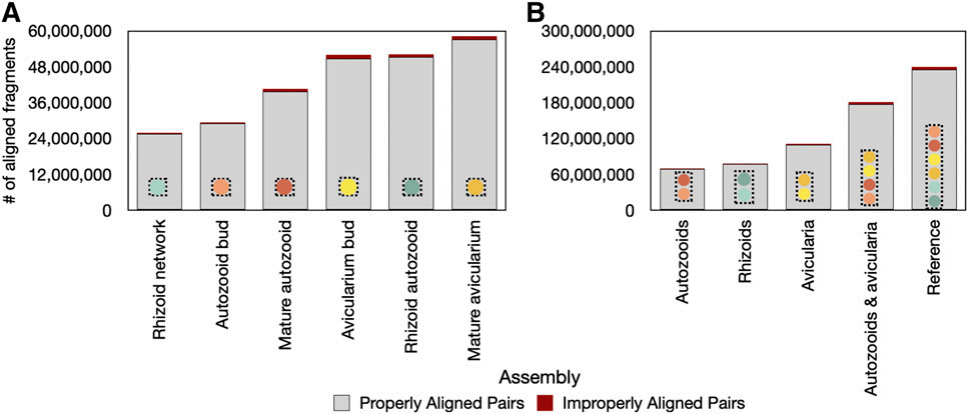
Read support for A) sample-specific and B) zooid-specific and combined transcriptome assemblies of *B. stolonifera*. Read pairs defined as ‘properly aligned’ mapped to the same contig and read pairs defined as ‘improperly aligned’ mapped to two different contigs. ’Autozooids’ = autozooid buds and mature autozooids, ’Rhizoids’ = rhizoid autozooids and rhizoid networks, ’Avicularia’ = avicularium buds and mature avicularia, ’Autozooids & avicularia’ = autozooid and avicularia, buds and mature stages.

Assemblies were determined to be largely complete. After searching each assembly for 978 total metazoan BUSCOs, we uncovered anywhere from 77.7 to 97.4% complete BUSCOs, between 1.5–19.2% fragmented BUSCOs, and from 0.9–3.1% missing BUSCOs (Figure 5). Of the complete BUSCOs in each assembly, 22.5–61.6% were classified as single-copy and 22.5–72.5% were classified as duplicated (Fig S4), so the search for BUSCOs was repeated on a subset of each assembly that included only the longest isoform representative of each gene. Assembly completeness remained high (74.3–97.2%) and of these complete BUSCOs in each assembly subset, 69.9–90.1% were classified as single-copy and only 2.0–7.1% were classified as duplicated (Fig. S5). Read support for each assembly was high, as 97.4–98.3% of aligned reads for each assembly mapped properly back to transcriptome contigs (Figure 6).

### Differential gene expression analysis

**Figure 7:**
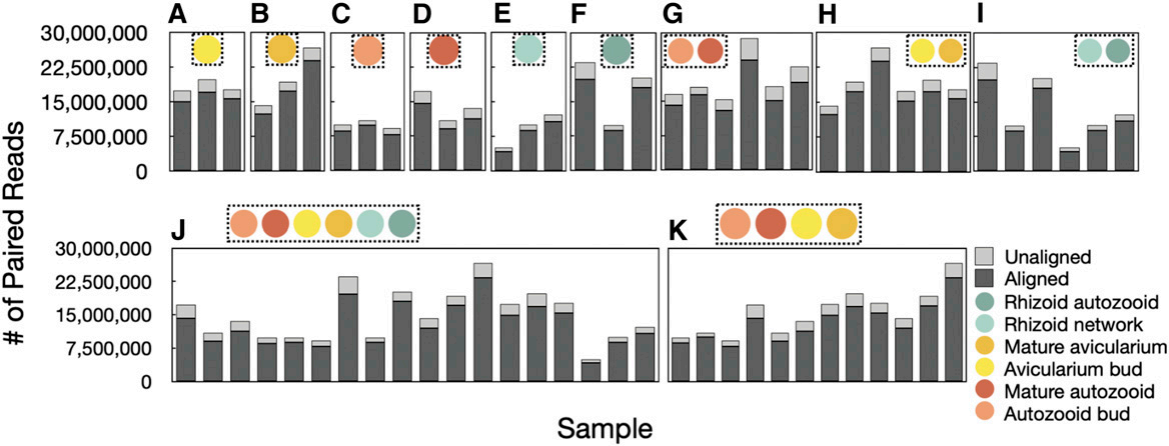
Assembly-guided read mapping using RSEM for tissue-specific assemblies, A-F) Avicularium buds, Mature avicularia, Autozooid buds, Mature autozooids, Rhizoid networks, Rhizoid autozooids, and sample-specific assemblies G-K) Autozooids, Avicularia, Rhizoids, Reference, Autozooids and Avicularia. Unaligned reads are defined as one or both reads in a pair not mapping to the assembly, for aligned reads, both read pairs align.

After building gene expression and count matrices for each assembly with RSEM, read alignment rates ranged from 82.8–91.7% (with an average alignment rate of 86.4% ± 0.3%, SE) (Figure 7). Using expression matrices generated from these mapped reads, ExN50 metrics were calculated for each assembly. ExN50 is suggested to be an accurate measure of assembly quality, that takes only into account the highest transcript expression (Haas *et al.*, 2013). The average ExN50 of our transcriptome assemblies was 1.433 kb (± 0.089 kb, SE) with an average of 57,316 transcripts (± 3,684, SE) (Table 3, Fig. S6). Subsequent differential expression analyses of zooid type and developmental stage within the reference transcriptome yielded a large number of significantly DE genes in comparison between autozooid and avicularium tissue.

**Table 3:** E90N50 Calculation for each assembly. E90N50 is calculated as the contig N50 (the size of contigs where half of the assembled bases are found) that is limited to the transcripts that represent 90% of the total normalized expression data

Assembly	# kb	# Transcripts
Autozooid Bud	1.286	49,363
Mature Autozooid	1.510	50,491
Avicularium Bud	1.042	48,463
Mature Avicularium	1.504	79,439
Rhizoid Autozooid	1.471	56,151
Rhizoid Network	1.095	50,844
Avicularia	1.471	82,264
Reference	1.943	47,714
Rhizoids	1.507	57,112
Autozooids	1.095	50,844
Autozooids & Avicularia	1.841	49,793

### Reference transcriptome

**Figure 8:**
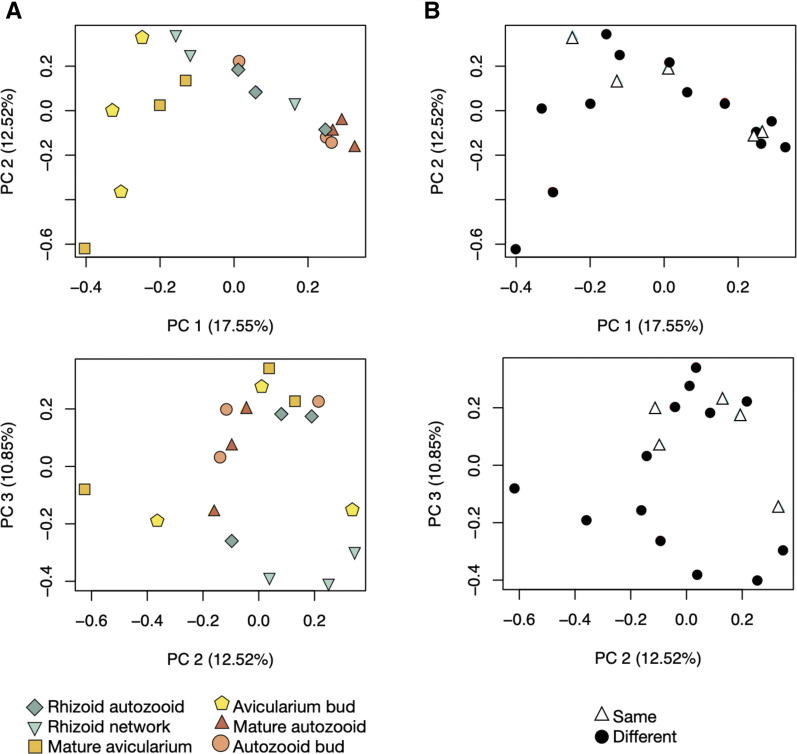
Reference transcriptome sample relationships. Principal components analysis (plotted as PC1 *vs.* PC2 and PC2 *vs.* PC3) of each sample in the log2 gene count matrix of the reference transcriptome assembly. A) Samples are labeled by tissue type and developmental stage. B) Samples are labeled by genetic individual, where samples labeled ‘Different’ originate from genetically distinct colonies and samples labeled ‘Same’ represent tissue samples of different zooid type and stage that originated from the same genetic individual.

In the principal components analysis exploring the similarity between the reference transcriptome assembly samples, samples of the same zooids obtained from different colonies tended to group more closely than samples of different zooids obtained from the same colony (Figure 8A). Along PC1 (which explains 17.55% of the variation in the data) samples tended to sort broadly by zooid type with autozooid samples around 0.2, avicularia samples around -0.4 and rhizoid samples spanning the space between the two groupings, around 0.0. PC2 (responsible for explaining 12.52% of the data) showed tighter clustering of autozooids (around 0.0) than avicularia (spanning from -0.6 to 0.3). When this same PCA plot is labeled by genetic individual (highlighting the biological replicate for each sample type that originated from the same colony contrasted with the remaining samples which originated from different colonies) (Figure 8B), we see on PC1 that the samples originating from the same colony range from -0.3 to 0.2 and occupy a similar space on this axis as the samples originating from different colonies. On PC2 we see that samples originating from the same colony occupy space on half of the y axis, from -0.1 to 0.3, whereas sample from different colonies range from -0.6 to 0.3, suggesting that there could be some small impact of genetic identity on colony similarity.

**Figure 9:**
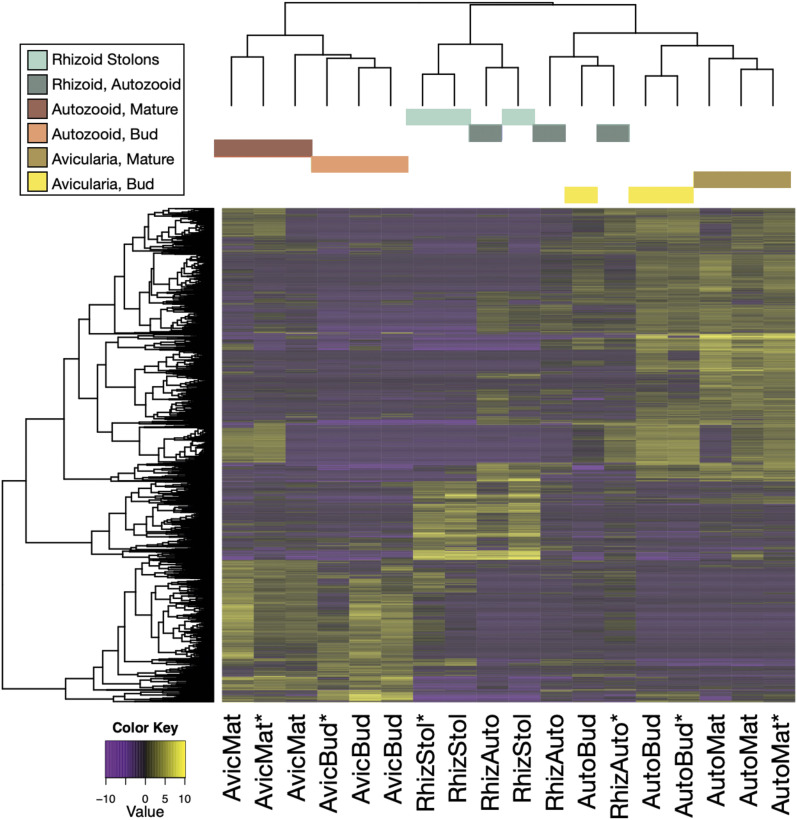
Reference transcriptome differentially expressed features. Significantly differentially expressed gene-level features (at a fourfold or greater level, FDR P *<* 0.001) are plotted by sample (1,597 genes, total). The color key corresponds to expression values which are log2-transformed and median-centered by gene.

**Figure 10:**
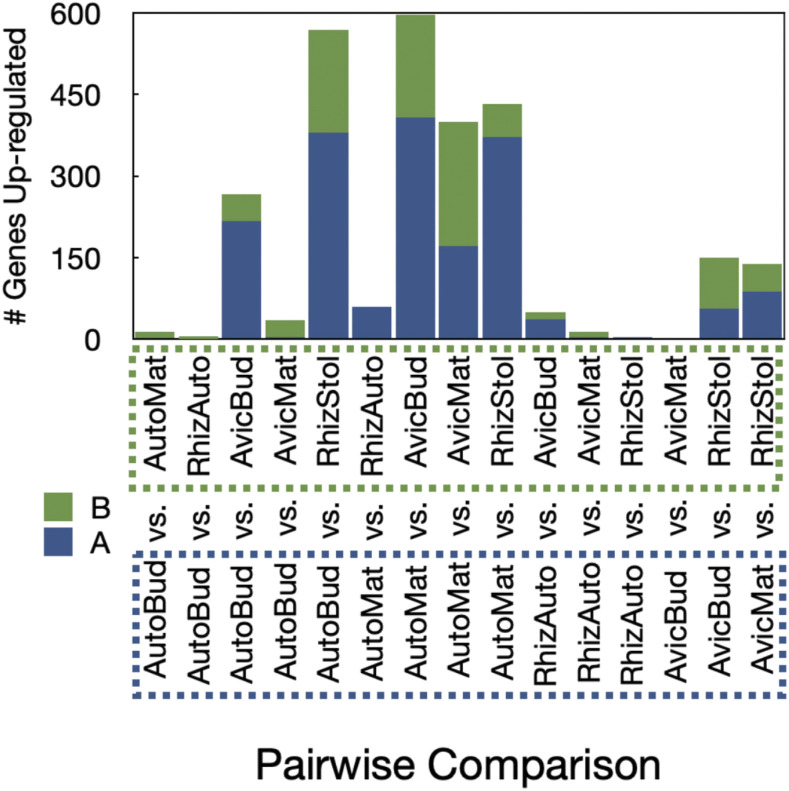
Reference transcriptome up-regulated genes for each pairwise comparison. Numbers of significantly upregulated genes (at a fourfold or greater level, FDR p *<* 0.001) are plotted for representatives of each pairwise contrast on the y-axis. Up-regulated genes in the first representative of the contrast are shown in graph as blue and x-axis bounded by dashed blue line and up-regulated genes in the second representative of the contrast are shown in the graph as green, and on the x-axis bounded by a dashed green line. Sample abbreviations are as follows: ‘AutoBud’ = autozooid bud, ‘AutoMat’ = mature autozooid, ‘AvicBud’ = avicularium bud, ‘AvicMat’ = mature avicularium, ‘RhizAuto’ = rhizoid autozooid, and ‘RhizStol’ = rhizoid network.

A total of 1,597 genes were differentially expressed in the reference transcriptome (Figure 9). An analysis of these DE up-regulated genes by pairwise comparison suggests that the greatest differential expression occurs in autozooid and avicularium contrasts (such as ‘AutoBud *vs.* AvicBud’, ‘AutoMat *vs.* AvicBud’, and ‘AutoMat *vs.* AvicMat’) and in comparisons between rhizoid networks and autozooids (such as ‘AutoBud *vs.* RhizStol’ and ‘AutoMat *vs.* RhizStol’) (Figure 10). While the majority of differentially expressed transcripts are unique to individual zooids (465 unique transcripts found in mature autozooids, 240 unique transcripts found in autozooid buds, 226 unique transcripts found in the rhizoid network, and 191 unique transcripts found in the mature avicularium, and 134 unique transcripts found in the autozooid bud), some transcripts were unique to subsets of related tissues, such as 150 transcripts unique to mature autozooids and autozooid buds, 105 transcripts unique to avicularium buds and mature avicularia, and 23 transcripts unique to rhizoid autozooid, autozooid bud, and mature autozooid tissue (Fig. S7A, S7B, Heberle *et al.* 2015; Conway *et al.* 2017).

### Autozooid & avicularium transcriptome

In order to further explore differential gene expression between autozooid and avicularium tissue, we analyzed sample relationships and differential expression within the avicularium and autozooid transcriptome (constructed from autozooid bud, mature autozooid, avicularium bud, and mature avicularium samples). A principal components analysis explored similarity relationships between the samples that contributed to this transcriptome to contrast similarity due to genetic individuality and similarity due to zooid type. Samples of the same zooid type tended to group

together closely (Fig. S8A) along PC1, which explains 23.48% of the variation in the data, with autozooid samples around 0.2 and avicularia samples between -0.1 and -0.4. On PC2 (15.87% of the data) and PC3 (12.08% of the data) autozooid samples group tightly, whereas avicularia samples are more dispersed. In contrast, when looking at the same PCA plot, labeled by tissues obtained from the same and different colonies (Fig. S8), there is a much less distinct grouping of samples by label, except for two autozooid samples, which can be found grouped together very closely for all three axes of PC1, PC2 and PC3.

**Figure 11:**
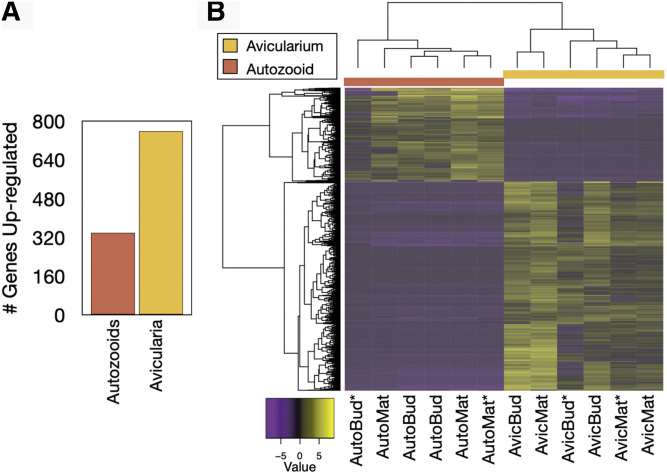
Autozooid and avicularium transcriptome differentially expressed features. Significantly differentially expressed gene-level features (at a fourfold or greater level, FDR p *<* 0.001) are plotted by sample (1,097 genes total). A) Number of significantly up-regulated genes in autozooids and avicularia samples. B) Samples *vs.* features map of diferentially expressed genes. Asterisks indicate the biological replicate that originated from the same genetic individual). The color key corresponds to expression values which are log2-transformed and median-centered by gene.

**Figure 12:**
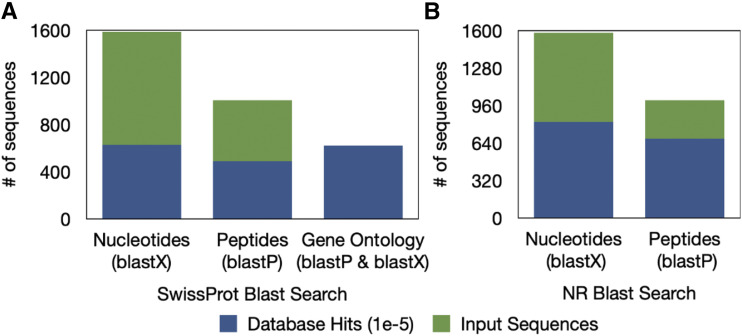
Autozooid and avicularium transcriptome functional annotation of the subset of differentially expressed genes, using the A) SwissProt database (to extract GO terms) and the B) NCBI ‘NR’ protein database. Number of sequences input, and number of sequences with a database hit are plotted for BLASTx searches of nucleotides (from each transcriptome assembly) and BLASTp searches of peptides (from longest open reading frame sequences predicted from the transcriptome assembly by Transdecoder [Haas *et al.*, 2013]).

We found 1,097 genes to be DE in the autozooid and avicularium transcriptome, with 339 genes up-regulated in autozooids and 758 genes up-regulated in the avicularia (Figure 11). Of these transcripts, 51.9% of nucleotides and 67.6% of predicted longest open-reading-frame peptides were annotated with unique database hits, after blasting to NCBI’s ‘NR’ protein database (E-value *<* 1e-5) (Figure 12).

A gene ontology enrichment analysis was performed on this subset of DE transcripts using the SwissProt database (E-value *<* 1e-5) and identified GO terms for 39% of transcripts. Gene ontology enrichment analysis showed autozooid biological process GO categories enriched (*P* < 0.05) in categories such as negative regulation of cytoplasmic translation, chondrocyte morphogenesis, response to nitrate, and ethanol catabolism, and avicularium biological process GO categories enriched (*P* < 0.05) in categories such as cytoplasmic translation, actin filament-based processes, hydrogen ion transmembrane transport, and response to light intensity (Fig. S9). GO enrichment analysis showed enrichment of cellular component GO terms in autozooids such as eukaryotic translation initiation factor, acrosomal membrane, and collagen type VI trimer; and in avicularia such as ribosome, macromolecular complex, extracellular matrix, organelle, and plasmodesmata (Fig. S10).

In the same GO analysis, in autozooids, we found enriched GO terms in the molecular function category such as NAD+ activity, translation repressor activity, nucleic acid binding, and acylcarnitine hydrolase activity, and in avicularia, we found enriched GO terms such as structural constituent of ribosome, structural molecule activity, rRNA binding, metal cluster binding, and unfolded protein binding (Fig. S11).

## Discussion

In this study we present the first characterizations of differential gene expression between different polymorphic zooids of a bryozoan colony. Although some researchers have sequenced bryozoan genes for systematic studies (*e.g.*, Fuchs *et al.* 2009; Waeschenbach *et al.* 2012; Laumer *et al.* 2019), development and metamorphosis studies (Wang *et al.* 2010; Wong *et al.* 2012, 2014), and studies of a bacterial symbiont (Mathew and Lopanik 2014), the application of high-throughput sequencing technologies is just beginning to be explored in this animal phylum.

By culturing bryozoans in the lab, the risk of sequence contamination by epibionts, a common source of sequencing error in the sequencing of fouling community organisms (Waeschenbach *et al.* 2012; Nesnidal *et al.* 2013), was minimized. High quality mRNA was extracted from zooids of individual lab-cultured colonies to construct 18 cDNA libraries (Table 1). Due to the fact that bryozoan specimens were fed exclusively on one food source in their lifetime (a single strain of *R. salina*), we were also able to isolate and address another potential source of sequence contamination, by removing reads that mapped to a transcriptome of that food source (Fig. S4). Removing potential contaminant reads prior to a transcriptome assembly reduces the potential formation of chimeric contigs (Vijay *et al.* 2013) and minimizes the chances of false positives in differential expression analyses.

### Assembly quality

We assembled eleven *de novo* transcriptomes of *Bugulina stolonifera* zooids (Table 2) for which transcript counts and contig N50 values are similar to those of other published bryozoan transcriptome assemblies (Wong *et al.* 2014; Laumer *et al.* 2019). Sample-level assemblies (n = 3 biological replicates) were largely complete, with 77–92% of complete BUSCOs recovered from a search of 978 single-copy orthologs, with slightly higher fragmentation in the avicularium bud and rhizoid network samples (19.2% and 16.7%, respectively) when compared to the remaining samples (which had between 3.9% and 7.1% fragmented BUSCO’s). When samples were grouped together into zooid-level assemblies, BUSCO completeness was remarkably high, ranging from 95.2 to 97.4%. These values are especially notable, as none of the sequences in the *metazoa odb9* BUSCO database have bryozoan origin (Waterhouse *et al.* 2017). Of the complete BUSCOs recovered in our analyses, a large percentage were defined as duplicated, which may be due to their presence of multiple isoforms. Once the transcriptome datasets were reduced to contain only the longest isoform representative per gene, BUSCO completeness remained high and the percentage of duplicated BUSCOs decreased to only a small fraction of those complete BUSCOs (Fig. S5). Quality metrics (BUSCO completeness, read support, and ExN50 value; Figures 5–6, Table 3) indicate that high-quality data were obtained from each sample of zooid type and stage.

### Differential gene expression

Analyses of differential gene expression were conducted within five tissue-specific assemblies. For the reference assembly (comprised of 3 biological replicates each, of autozooid buds, mature autozooids, avicularium buds, mature avicularia, rhizoid autozooids, and rhizoid network samples), pairwise comparisons between each of six different sample types were conducted, finding over 1,500 genes differentially expressed (Figure 9), with the highest differential expression found in comparisons between different zooid types, as opposed to within-zooid stage (Figure 10). For the combined autozooid–avicularium assembly (comprised of three biological replicates each of autozooid buds, mature autozooids, avicularium buds, and mature avicularium samples), the pairwise comparison between autozooid and avicularium samples yielded over a thousand DE genes, with over 300 genes up-regulated in autozooids and over 700 genes up-regulated in avicularia (Figure 11). These high numbers of DE genes between different zooid types in both the reference transcriptome and the grouped autozooid–avicularium transcriptome appear to indicate unique genetic programs involved with the growth and maintenance of different zooid polymorphs within a colony (Table S3), with the caveat that some of these observed expression differences may instead be attributed to the increased numbers of PCR cycles used for amplification of avicularium samples. Genes involved in the Wnt and Notch signaling pathways were found to be significantly up-regulated in both autozooid tissue (*RNF213*, *nphp3*, and *DTX3*) and in avicularium tissue (*Rack1* and *Notch1*). Genes involved with muscular development (*APOD*, *blc*, *elF4E1*, *Rac1*, *tcaf*, *Tnc*, *Ttn*, *emb-9*) and heat response (*CKM*, *HSP70*, *HSP90-2*) were found to be significantly up-regulated in avicularium tissue. Genes involved in neuron development were found in both avicularium (*TENM1*, *tenm3*,) and autozooid (*Cel*, *Matn2*, *FBXO39*, *CPEB1*, *RpL10*) tissue.

These results are difficult to compare with other studies on polymorphic colonies, as few have addressed differential gene expression across polymorphs. From a methodological context, siphonophores have been used as a model to compare assembly techniques (Siebert *et al.* 2011), and other studies have focused on examining different life stages in the biphasic life cycle of a hydrozoan (Sanders and Cartwright 2015). Even with bryozoans, levels of expression have been studied to select candidate developmental genes for body patterning (especially the lophophore and digestive tract) (Wong *et al.* 2014), but this study compared larval and whole-colony data, without discerning among polymorphic zooids, as done here. Thus, to our knowledge this is the first study attempting to truly quantify and explore DGE among polymorphs of a colonial metazoan. Ultimately, comparing the genetic processes behind establishing division of labor in bryozoans to those in other colonial taxa may help reveal clues about the enigmatic evolution of polymorphism in some colonial animals (Hiebert *et al.* 2020) and more broadly, the processes involved in the evolution of colonial animals in general (Simpson *et al.* 2020).

### Transcriptome annotations

The relatively high percentage of unannotated sequences in our transcriptome is likely due to the low representation of bryozoan sequences in publicly available databases and percent annotation in this study is similar to those in studies of other underrepresented metazoans (Wong *et al.* 2014; Kitchen *et al.* 2015; Álvarez-Campos *et al.* 2019).

Based on the anatomical differences between autozooids and avicularia (Carter *et al.* 2010b 2011), we anticipated finding genes involved in development of autozooid-specific anatomical structures such as those associated with sexual reproduction, feeding, and digestion to be up-regulated in autozooids when compared to avicularia. Among the 39% of DE transcripts with assigned GO terms for this comparison, we found GO terms up-regulated in autozooids for GO categories involved with muscular, exocrine, and nervous system development, response to chemical stimuli, and metabolic processes (Fig. S9A). Additionally, we uncovered a suite of transcripts involved in development and regeneration that may be related to the formation of autozooid buds or involved in the repeated lifelong cycle of degeneration and regeneration undertaken by the bryozoan autozooid polypide, in a process known as brown body formation (Gordon 1977) or polypide recycling. During this process, polypides periodically degenerate to produce a brown body and are replaced by a new polypide within the same zooid, in some cases completing up to five polypide cycles (Barnes and Clarke 1998; Giribet and Edgecombe 2020).

Although the exact function(s) of avicularia remain largely unproven, avicularia have been hypothesized to play a defensive role within the bryozoan colony by providing mechanical defense (Winston 1986, 1991) as well as chemical deterrence (Carter *et al.* 2010b; Schack *et al.* 2019) and sensory abilities (Winston 1991; Schack *et al.* 2019). Based on observations of large pedunculate avicularia moving independently from the colony and responding to mechanical stimuli (Darwin 1872; Ryland 1960; Kaufmann 1968), we expected to find genes involved in the development of musculature as well as genes involved with sensing and responding to the environment and secretion to be up-regulated in avicularia when compared to autozooids. Transcripts were up-regulated in avicularia for GO term categories such as anatomical development, response to stimuli, as well as specific terms related to pharyngeal pumping, energy taxis, growth, and autophagy (Fig. S9B). We also found up-regulated anatomical developmental transcripts (including GO terms for the development of hemocytes, epithelial cells, and peripheral nervous system axons) and stimulus-response transcripts that could be associated with a defensive role (including response to light intensity, cadmium, abiotic stimuli, hormones, heat, osmotic stress, and wounding). While avicularia do not have an active reproductive or digestive system, they do have a vestigial polypide, as they are derived autozooids (Kaufman 1971; Carter *et al.* 2010b 2011). Interestingly, several upregulated transcripts in avicularia are involved in metabolic processes (including GO terms for cellular macromolecule metabolic processes, nitrogen compound metabolism, and mucilage metabolism) and reproduction (GO terms for spermatocyte division, development of male secondary sex characteristics, male germ line-cyst formation, male meiosis chromosome segregation, and developmental process involved in reproduction) (Fig. S9). Based on our understanding of avicularian anatomy, this genetic signal of metabolism may be due to the metabolism of nutritional products transported from feeding autozooids into the avicularium via the funiculus. It is unlikely, however, that the genetic signal of reproduction is coming from the avicularium itself and is more likely coming from peduncle cushion tissue that was included during the dissection of avicularia, although this cannot be discerned with the current experimental design. The peduncle cushion forms as an out-pocketing of the wall of the autozooid (Kaufman 1971) and presumably could include tissue from the funiculus and transient testes of the parental autozooid (in bryozoans, gametes usually arise from transient patches of germinal tissue associated to the funiculus), which are found in close association along the autozooid body wall Carle and Ruppert (1983).

Many of the transcripts uncovered have sensory and muscular functions (*APOD*, *rhoaa*, *Mov10|1*, *blc*, *Rac1*, *tcaf*, *Rnc*, *Ttn*, *emb-9*) which is in accordance with previous published descriptions of avicularia being highly muscular structures with mechanical and chemical sensory capabilities (Kaufman 1971; Mukai *et al.* 1997; Carter *et al.* 2010a,b; Lidgard *et al.* 2011). Interestingly, the gene ontology analysis yielded some unexpected categories of DE transcripts in avicularia, which sets the groundwork for future testing of novel gene-expression driven hypotheses about avicularium behavioral response to chemicals, light, and heat (*HSP90-2*, *CKM*, *HSP70*, *HSP70-4*). While there has been some documentation of avicularium responses to chemical stimuli (Winston 1984, 1991), many open questions remain. To the best of our knowledge, the sensory responses of avicularia to both light and heat cues have not been formally assessed.

One challenge inherent to this study and future transcriptomic investigations of bryozoan polymorphic zooids is the differing amounts of starting material across samples, in particular, the limited amount of tissue in the bud form of the avicularium. The authors, limited by sequencing technology, used additional rounds of PCR cycles to amplify this tissue prior to sequencing, which resulted in the potential downstream loss of signal from lowly expressed genes in the avicularium samples (both bud and avicularium stage) due to amplification bias. For this reason, the authors have focused their analysis on transcript presence rather than transcript abundance. The authors recommend that future studies take advantage of modern single-cell sequencing technologies to equally leverage input from small and large zooid types alike, in order to enhance transcript presence and abundance comparisons.

An exciting next step for this study of bryozoan gene expression would involve conducting functional genetic assays comparing the location and level of expression of certain genes of interest in developing autozooids and avicularia to quantify and corroborate this DGE data. It would be particularly interesting to further explore the roles and localization of Wnt, and Notch signaling pathway genes, which we have discovered to be significantly expressed in both autozooids and avicularia. Wnt, BMP, Notch, and Hedgehog signaling pathway genes have been previously identified as playing a role in the formation of the lophophore and digestive tract of the ancestrula of *Bugula neritina* (Wong *et al.* 2014), however it would be interesting to further understand how these genes are involved in the formation of structures within developing autozooids and avicularia. An additional next step would involve a comparative study, contrasting the zooid and developmental stage-specific DGE patterns of *B. stolonifera* to those of other cheilostome species. This could help to identify broader gene expression patterns that are associated with the formation and maintenance of polymorphic zooids that are conserved across cheilostome bryozoans, or to identify expression patterns involved in the formation of polymorphic zooids that are unique to different lineages of cheilostomes.

Currently, the ability to functionally annotate bryozoan genes is limited by the taxonomic scope of publicly available databases. Additionally, GO term assignments can be problematic for organisms like bryozoans that are poorly annotated (Škunca *et al.* 2017). While it was possible to assign functional annotations and GO term assignments to some DE genes with SwissProt and NR databases, over half of the DE genes we identified have no known function or identity. As the cost of sequencing decreases and RNA-sequencing and genomics become more accessible to invertebrate zoologists who study bryozoans and other less-well represented metazoans, taxonomic diversity of publicly available databases will improve.

This study provides the first analysis of DGE between zooids within a bryozoan colony and provides a foundation for future analyses of similar comparisons in other species, particularly with respect to the evolution, development, and function of the bryozoan avicularium, but the techniques and approaches can be extended to other bryozoan heterozooids or to other colonial organisms. The methodologies we have presented for extracting high-quality mRNA from zooids with low contamination, together with the resulting DGE dataset, provide a new approach for exploring long-unanswered questions about the function, evolution, and development of polymorphic zooids within bryozoan colonies. These results will aid in future annotations of bryozoan transcriptome and genomes studies, which will provide deep and meaningful insights into the longstanding questions about evolution and establishment of division of labor in bryozoans.
